# Administration of L-Type Bovine Spongiform Encephalopathy to Macaques to Evaluate Zoonotic Potential

**DOI:** 10.3201/eid3105.241257

**Published:** 2025-05

**Authors:** Morikazu Imamura, Ken’ichi Hagiwara, Minoru Tobiume, Minako Ohno, Hiromi Iguchi, Hanae Takatsuki, Tsuyoshi Mori, Ryuichiro Atarashi, Hiroaki Shibata, Fumiko Ono

**Affiliations:** University of Miyazaki, Miyazaki, Japan (M. Imamura, M. Ohno, H. Iguchi, H. Takatsuki, T. Mori, R. Atarashi); National Institute of Infectious Diseases, Tokyo, Japan (K. Hagiwara, M. Tobiume); The Corporation for Production and Research of Laboratory Primates, Tsukuba, Japan (H. Shibata); Okayama University of Science, Imabari, Japan (F. Ono)

**Keywords:** prions, zoonoses, oral transmission, bovine spongiform encephalopathy, cynomolgus macaques, L-Type bovine spongiform encephalopathy, *Macaca fascicularis*, Japan

## Abstract

We administered L-type bovine spongiform encephalopathy prions to macaques to determine their potential for transmission to humans. After 75 months, no clinical symptoms appeared, and prions were undetectable in any tissue by Western blot or immunohistochemistry. Protein misfolding cyclic amplification, however, revealed prions in the nerve and lymphoid tissues.

Worldwide emergence of classical bovine spongiform encephalopathy (C-BSE) is associated with variant Creutzfeldt-Jakob disease in humans (*1*). Two other naturally occurring BSE variants have been identified, L-type (L-BSE) and H-type. Studies using transgenic mice expressing human normal prion protein (PrP^C^) (*2*) and primates (*3*–*5*) have demonstrated that L-BSE is more virulent than C-BSE. Although L-BSE is orally transmissible to minks (*6*), cattle (*7*), and mouse lemurs (*5*), transmissibility to cynomolgus macaques, a suitable model for investigating human susceptibility to prions, remains unclear. We orally inoculated cynomolgus macaques with L-BSE prions and explored the presence of abnormal prion proteins (PrP^Sc^) in tissues using protein misfolding cyclic amplification (PMCA) along with Western blot (WB) and immunohistochemistry (IHC). PMCA markedly accelerates prion replication in vitro, and its products retain the biochemical properties and transmissibility of seed prion strains (*8*).

## The Study

Two macaques orally inoculated with L-BSE prions remained asymptomatic and healthy but were euthanized and autopsied at 75 months postinoculation. WB showed no PrP^Sc^ accumulation in any tissue (Table), IHC revealed no PrP^Sc^ accumulation, hematoxylin and eosin staining revealed no spongiform changes in brain sections, and pathologic examination revealed no abnormalities.

**Table:** Detection of PrPres in tissue samples obtained from macaques orally challenged with L-BSE prions in study of oral transmission of L-type bovine spongiform encephalopathy in macaques to evaluate zoonotic potential*

Sample location	PMCA, no. positive/no. total							WB, orally challenged animals			IHC, orally challenged animals	
	Orally challenged animals			Negative controls								
	18	19		9086	1004	9040		18	19		18	19
Nerve tissues												
Cerebral cortex, frontal lobe; PTA, PBS	0/2, 0/2	0/2, 0/2		ND	0/2, 0/2	0/2, 0/2		Neg	Neg		Neg	Neg
Cerebral cortex, temporal lobe	ND	ND		ND	ND	ND		Neg	Neg		Neg	Neg
Cerebral cortex, parietal lobe	ND	ND		ND	ND	ND		Neg	Neg		Neg	Neg
Cerebral cortex, occipital lobe	ND	ND		ND	ND	ND		Neg	Neg		Neg	Neg
Hippocampus	ND	ND		ND	ND	ND		Neg	Neg		Neg	Neg
Thalamus	ND	ND		ND	ND	ND		Neg	Neg		Neg	Neg
Cerebellum	ND	ND		ND	ND	ND		Neg	Neg		Neg	Neg
Cervical cord; EtOH, PBS	0/2, 0/2	0/2, **1/2**		0/2, 0/2	0/2, 0/2	0/2, 0/2		Neg	Neg		Neg	Neg
Thoracic cord; EtOH, PBS	0/2, **1/2**	0/2, **1/2**		0/2, 0/2	0/2, 0/2	0/2, 0/2		Neg	Neg		Neg	Neg
Lumbar cord; EtOH, PBS	0/2, 0/2	0/2, 0/2		0/2, 0/2	0/2, 0/2	0/2, 0/2		Neg	Neg		Neg	Neg
Median nerve, PTA	**2/2**	**1/2**		ND	ND	ND		Neg	Neg		Neg	Neg
Sciatic nerve, PTA	0/2	0/2		ND	ND	ND		Neg	Neg		Neg	Neg
Secondary lymphoid tissues												
Spleen, PTA	**1/2**	0/2		ND	ND	0/2		Neg	Neg		Neg	Neg
Tonsil, PTA	**1/2**	**2/2**		0/2	0/2	0/2		ND	ND		ND	ND
Submandibular lymph node, PTA	0/2	0/2		0/2	ND	0/2		Neg	Neg		Neg	Neg
Inguinal lymph nodes, PTA	**1/2**	**2/2**		ND	ND	ND		ND	ND		ND	ND
Mesenteric lymph node, PTA	**1/2**	**1/2**		0/2	0/2	0/2		ND	ND		ND	ND
Primary lymphoid tissues												
Thymus, PTA	0/2	**2/2**		ND	ND	ND		ND	ND		ND	ND
Others												
Submaxillary gland, PTA	**2/2**	**1/2**		ND	ND	ND		ND	ND		ND	ND
Ileum, PTA	**2/2**	**2/2**		0/2	0/2	0/2		Neg	Neg		Neg	Neg
*Bold indicates positive results. EtOH, ethanol precipitation; IHC, immunohistochemistry; ND, not determined; neg, negative; PBS, suspension in phosphate-buffered saline; PMCA, protein misfolding cyclic amplification; PrPres, PrP^Sc^-like proteinase K–resistant prion proteins; PTA, sodium phosphotungstic acid precipitation; WB, Western blot.												

**Figure 1:**
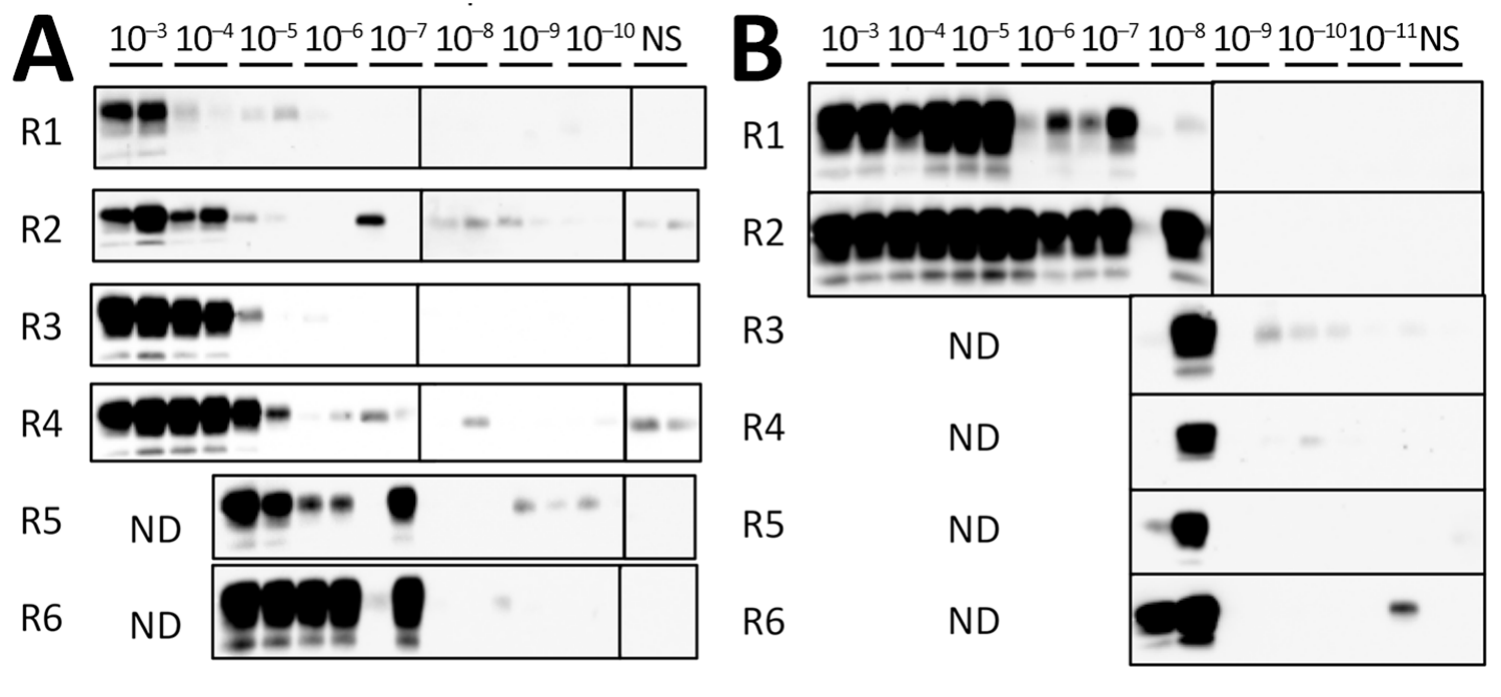
Sensitivity of modified protein misfolding cyclic amplification (PMCA) to detect abnormal prion protein in study of oral transmission of L-type bovine spongiform encephalopathy (L-BSE) in intracerebrally inoculated macaques to evaluate zoonotic potential. We evaluated in macaques intracerebrally inoculated with L-BSE prions (A) and cattle intracerebrally inoculated with classical BSE prions (B) (Appendix). We serially diluted (10^−3^–10^−11^) brain homogenates (10% weight by volume) in 50 μL of human normal prion protein (PrP^C^) substrate and performed PMCA. We further diluted the initial PMCA product to 1:5 with a fresh PrP^C^ substrate for subsequent rounds. We conducted 6 rounds of PMCA in duplicate. In macaques, PMCA propagated PrPSc-like proteinase K–resistant prion protein (PrP^res^) from a 10^−7^ dilution in the fifth amplification round; in cattle, PMCA propagated PrPres from a 10^−8^ dilution during the second amplification round. We performed Western blot for each PMCA product (2.5 μL) after proteinase K digestion using the T-2 antibody (*10*). ND, assays not done; NS, nonseeded control; R, round.

We next attempted to detect PrP^Sc^ using PMCA, performed as previously described (*9*), with minor modifications (Appendix). First, we evaluated the sensitivity of PMCA. Using serial amplification with 10-fold stepwise dilutions of prion-infected brain homogenates as seeds, we amplified PrP^Sc^-like proteinase K (PK)–resistant prion protein (PrPres) from a 10^−7^ dilution of 10% brain homogenate (BH) obtained from macaque intracerebrally inoculated with L-BSE prions in the fifth amplification round (Figure 1, panel A). This method also enabled propagation of PrPres from a 10^−8^ dilution of BH from C-BSE–affected cattle during the second amplification round (Figure 1, panel B), suggesting PMCA’s higher efficiency and sensitivity for detecting C-BSE prions than macaque L-BSE prions.

**Figure 2:**
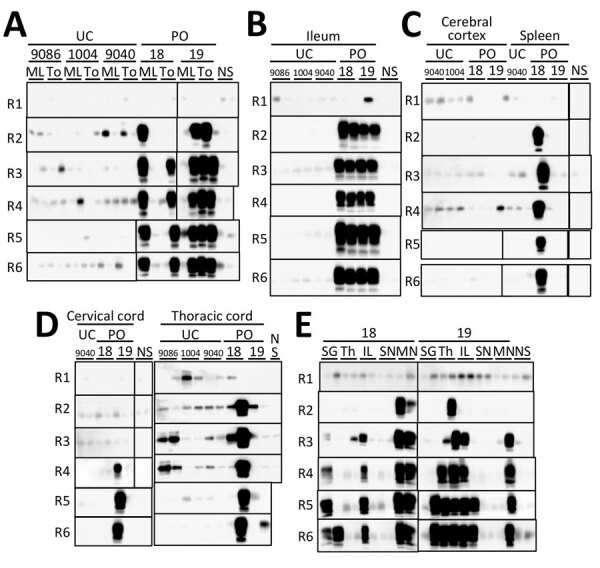
Sensitivity of modified protein misfolding cyclic amplification (PMCA) to detect abnormal prion protein in study of oral transmission of L-type bovine spongiform encephalopathy (L-BSE) in orally inoculated macaques to evaluate zoonotic potential. We performed 6 rounds of PMCA in duplicate in the tissues of 2 macaques (#18 and #19) orally inoculated with L-BSE prions, primarily in the lymphoid and nervous system tissues. A) ML nodes and To tissue; B) ileum; C) cerebral cortex and spleen; D) cervical and thoracic cords; and E) SG, Th, IL nodes, SN, and MN tissues. We prepared PMCA seeds, equivalent to 6.25 mg of tissue, obtained from lymphoid tissues, peripheral nerves, submaxillary glands, and ileum by using a standard sodium phosphotungstic acid precipitation method. For brain tissues, we used 10% homogenates in phosphate-buffered saline (250 μg) and phosphotungstic acid precipitates as seeds. For spinal cords, we used 10% homogenates in phosphate-buffered saline and ethanol precipitates (625 μg equivalent) as seeds. Tissues obtained from 3 uninfected macaques (#9068, #1004, and #9040) served as negative controls and were processed identically to those obtained from inoculated animals. For the thoracic spinal cord of macaque #19, we performed amplification for 7 rounds to determine whether the round 6 signal was positive. IL, inguinal lymph; ML, mesenteric lymph; MN, median nerve; NS, nonseeded control; PMCA, protein misfolding cyclic amplification; PO, L-type bovine spongiform encephalopathy orally inoculated macaques; SG, submaxillary gland; SN, sciatic nerve; TH, thymus; To, tonsil; UC, uninoculated control.

We attempted to detect prions in the lymphoid and nervous systems, among other tissues, of the 2 orally inoculated macaques using refined PMCA (Figure 2; Appendix Figure, Table). In lymphoid tissue samples prepared using sodium phosphotungstic acid precipitation (Appendix), we amplified PrPres in the inguinal and mesenteric lymph nodes, ileum, and tonsils of both macaques (Figure 2, panels A, B), as well as in the spleen of 1 macaque (#18) and the thymus of the other (#19), in the second or third amplification round of PMCA (Figure 2, panel C). We observed no PrPres in the submandibular lymph nodes (Appendix Figure 1). Examining the central nervous system, we observed no PrPres amplification in the cerebral cortex (Figure 2, panel C), whether seeded with phosphate-buffered saline homogenates or phosphotungstic acid precipitates. The spinal cord showed no PrPres amplification upon ethanol precipitation. However, PrPres was amplified in the cervical spinal cord of macaque #19 and in the thoracic spinal cord of both macaques with phosphate-buffered saline homogenates (Figure 2, panel D). We also confirmed PrPres in the median nerve of both macaques but not in the sciatic nerve (Figure 2, panel E). We noted PrPres signals in the submandibular glands of both animals. In contrast, we found no PrPres amplification in any tissues from uninoculated control macaques.

**Figure 3:**
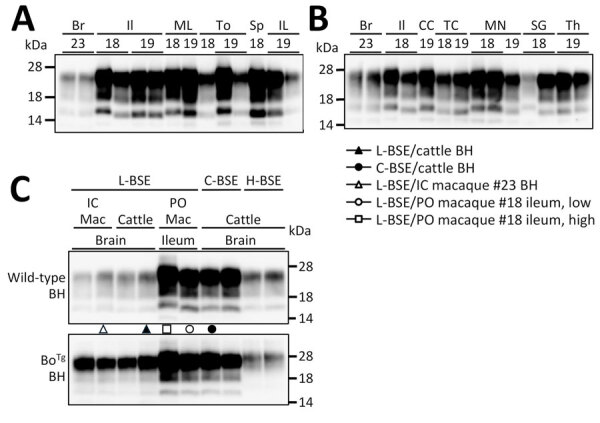
Gel electrophoresis and Western blot testing of tissues obtained from macaques orally inoculated with L-BSE prions in study of oral transmission of L-BSE in macaques to evaluate zoonotic potential. We loaded each 6th-round protein misfolding cyclic amplification (PMCA) product, seeded with tissues obtained from the 2 macaques (#18 and #19), onto 2 gels for sodium dodecyl sulfate-polyacrylamide gel electrophoresis, followed by Western blot. A) PMCA products amplified from ML, To, Sp, and Il; B) PMCA products amplified from CC, TC, MN, SG, and Th; C) Western blot analysis. For comparison, PMCA products amplified using BH obtained from macaque #23, intracerebrally inoculated with L-BSE, were run on both gels together with products amplified from the ileum of macaque #18. Abnormal prion protein (PrP)-like proteinase K–resistant prion proteins (PrPres) from L-BSE orally inoculated macaques exhibited a few distinct banding patterns, which differed from those of PrPres from L-BSE intracerebrally in macaque #23. For Western blot, we compared 6th-round PMCA products, seeded with IL tissue obtained from macaque #18 and Br tissue obtained from macaque #23, L-BSE–affected cattle, C-BSE–affected cattle, and H-BSE–affected cattle. Banding results represent the products of PMCA using the BHs of wild-type mice (upper image) and bovine normal prion protein–expressing transgenic mice (lower image) as substrates, with identical cofactors. Among the PrPres amplified from the ileum of L-BSE/PO macaque #18, the banding pattern of PrPres with a small molecular weight (low) was very similar to that of PrPres amplified from the brain of cattle inoculated intracerebrally with C-BSE. BH, brain homogenate, Bo^Tg^, bovine normal prion protein–expressing transgenic; Br, brain; C-BSE, classical bovine spongiform encephalopathy; CC, cervical cord; H-BSE, H-type bovine spongiform encephalopathy; IC, inoculated intracerebrally; Il, ileum; IL, inguinal lymph nodes; L-BSE, L-type bovine spongiform encephalopathy; Mac, macaque; ML, mesenteric lymph nodes; MN, median nerve; PO, inoculated orally; SG, submaxillary gland; Sp, spleen; TC, thoracic cord; Th, thymus; To, tonsil.

PrPres obtained from the orally inoculated macaques exhibited diverse banding patterns distinct from those generated by PMCA using L-BSE–affected cattle BH and L-BSE intracerebrally inoculated macaque BH as seeds (Figure 3, panels A–C). Of note, the lowest-molecular-weight PrPres variants from the ileum, spleen, inguinal lymph nodes, thoracic cord, submaxillary gland, and mesenteric lymph nodes of orally inoculated macaques exhibited remarkable PK resistance similarity and banding patterns indistinguishable from those of PrPres generated by PMCA with C-BSE–affected cattle BH as a seed (Figure 3, panel C, Appendix Figures 2 and 3). In contrast, the higher-molecular-weight PrPres variants from the ileum of macaque #18 exhibited a unique banding pattern distinct from those of L-BSE, C-BSE, and H-type BSE prions (Figure 3, panel C). Banding patterns and PK resistance of PrPres amplified from L-BSE–affected cattle BH and L-BSE intracerebrally inoculated macaque BH were notably similar.

This PMCA method was initially designed for the high-sensitivity detection of L-BSE intracerebrally inoculated macaque PrP^Sc^ but was even more efficient and sensitive in detecting bovine C-BSE PrP^Sc^ (Figure 1, panel B). Therefore, we believe that this method enabled the detection of both C-BSE–like PrP^Sc^ and potentially novel PrP^Sc^ variants.

## Conclusion

We noted no detectable evidence of PrP^Sc^ by WB or IHC in any tissues of L-BSE orally inoculated macaques. Nevertheless, PMCA successfully amplified PrPres from lymphatic and neural tissues. The PrPres exhibited electrophoretic patterns distinct from those detected by PMCA using L-BSE–affected cattle BH as the seed (Figure 3, panel C), indicating that the PrP^Sc^ used as the template for PrPres amplification in orally inoculated macaques did not originate from the bovine L-BSE prions used as inoculum. Instead, PrP^Sc^ were newly generated by the conversion of macaque PrP^C^ by bovine L-BSE prions. Our results provide strong evidence that L-BSE can infect macaques via the oral route.

We found no evidence that PrP^Sc^ reached the brain in orally inoculated macaques; however, the macaques euthanized 6 years postinoculation might have been in the preclinical period. At low infection levels, lymph nodes play a vital role in prion spread to the central nervous system (*11*). Therefore, had the macaques been maintained for a longer period, they might have developed prion disease. Retrospective surveillance studies using the appendix and tonsil tissues suggested a considerable number of humans harboring vCJD in a carrier state (*12*). Thus, we cannot exclude that L-BSE orally inoculated macaques could similarly remain in a potentially infectious state.

The brain of L-BSE intracerebrally inoculated macaque accumulated prions with biochemical properties resembling bovine L-BSE prions (Figure 3, panel C; Appendix Figure 2); however, we observed no PrP^Sc^ accumulation in lymphoid tissues by WB or IHC (*4*). In contrast, macaques orally inoculated with C-BSE prions showed PrP^Sc^ accumulation in lymphoid tissues, including the spleen, tonsils, and mesenteric lymph nodes by WB and IHC (*13*). In our study, L-BSE orally inoculated macaques harbored C-BSE–like prions in their lymphoid and neural tissues. Interspecies transmission of L-BSE prions to ovine PrP transgenic mice can result in a shift toward C-BSE–like properties (*14**,**15*). Our data suggest that L-BSE prions may alter biophysical and biochemical properties, depending on interspecies transmission and inoculation route, acquiring traits similar to those of C-BSE prions. This transformation might result from structural changes in the L-BSE prion to C-BSE–like prions and other lymphotropic prions within lymphoid tissues or from the selective propagation of low-level lymphotropic substrains within the L-BSE prion population.

The first limitation of our study is that the oral inoculation experiment involved only 2 macaques and tissues collected at 6 years postinoculation, before disease onset. Consequently, subsequent progression of prion disease symptoms remains speculative. A larger sample size and extended observation periods are required to conclusively establish infection in orally inoculated macaques. Furthermore, we performed no bioassays for PMCA-positive samples, leaving the relationship between PMCA results and infectious titers undefined. Considering that PrPres amplifications from tissues from the orally inoculated macaque tissues required 2 rounds of PMCA, the PrP^Sc^ levels in positive tissues might have been extremely low and undetectable in the bioassay.

Previous studies have demonstrated that L-BSE can be orally transmitted to cattle (*7*) and might have caused prion disease in farm-raised minks (*6*), indicating that L-BSE could naturally affect various animal species. Our findings suggest that L-BSE can also be orally transmitted to macaques. Therefore, current control measures aimed at preventing primary C-BSE in cattle and humans may also need to consider the potential risk of spontaneous L-BSE transmission.

Appendix Additional information for oral transmission of L-type bovine spongiform encephalopathy in macaques to evaluate zoonotic potential
