# Supplementary material for: Administration of L-Type Bovine Spongiform Encephalopathy to Macaques to Evaluate Zoonotic Potential
Source: Emerg Infect Dis. 2025 May;31(5):986–90. doi: 10.3201/eid3105.241257 (PMC12044238; doi:10.3201/eid3105.241257)
Supplement: Appendix — Additional information for oral transmission of L-type bovine spongiform encephalopathy in macaques to evaluate zoonotic potential [file 24-1257-Techapp-s1.pdf]

Article DOI: http://doi.org/10.3201/eid3105.251257

EID cannot ensure accessibility for supplementary materials supplied by authors. Readers who have difficulty accessing supplementary content should contact the authors for assistance.

Oral Transmission of L-type Bovine Spongiform Encephalopathy in Macaques to Evaluate Zoonotic Potential

Appendix

Methods

Animal Experiment

The animal experiments were approved by the Animal Welfare and Animal Care and Use Committee (approval ID: DS23–41) and the Animal Ethics Biosafety Committee (approval ID: BSL3-R-10.04, BSL3-R-11.09, and BSL3-R-12.07) at the National Institutes of Biomedical Innovation, Health, and Nutrition, Japan. Two macaques, ≈1.2-year-old (#18 and #19), were orally inoculated with 8 g (5 mL of 20% homogenate for 8 times) of brain tissue from a natural L-BSE case in Japan (case #24).

The two macaques remained asymptomatic and healthy but were euthanized and autopsied at 75 months post-inoculation. For ethical considerations, we established a 75-month endpoint for the prion infection study in cynomolgus macaques. This decision was informed by prior findings that cynomolgus macaques inoculated with brain homogenates (BHs) from L-BSE-infected cattle manifested clinical signs approximately 2 years post-inoculation (*3*) and aimed to balance scientific inquiry with animal welfare considerations.

Uninoculated macaques (#9068 and #1004, #9040) were euthanized at 7 years and 6 months, 7 years and 2 months, and 22 years and 3 months, respectively, served as negative controls.

Western Blot (WB) Analysis of Tissues from Macaques Orally Inoculated with L-BSE Prion

Brain homogenates (50 µL, 20% w/v in PBS) of macaques #18 and #19 were mixed with 50 µL of a buffer containing 4% zwittergent 3–14, 1% lauroylsarcosine, 0.5 mg/mL collagenase, 60 unit/mL benzonase, 100 mM NaCl, and 50 mM HEPES-NaOH (pH 7.4), and incubated at 37°C for 30 minutes. Proteinase K (PK) was added to a final concentration of 50 µg/mL, and samples were incubated at 37°C for 45 minutes. After adding 50 µL of 2-butanol/methanol (5:1, v/v) with 10 mM PMSF, the mixture was centrifuged at 18,000 xg for 15 minutes at 18°C. Pellets were air-dried and stored at −75°C.

Non-brain tissues were similarly treated, then resuspended in 100 µL of buffer (2% zwittergent 3–14, 0.5% lauroylsarcosine, 100 mM NaCl, and 50 mM HEPES-NaOH, pH 7.4) and digested with 2.5 µg/mL PK at 37°C for 30 minutes before PMSF treatment and centrifugation.

PK-digested samples were analyzed by sodium dodecyl sulfate (SDS)- polyacrylamide gel electrophoresis (PAGE) and western blot using Invitrolon PVDF membranes. Membranes were blocked with PBS containing 0.1% Tween 20 and 4% fetal calf serum, incubated with the anti-PrP 3F4 antibody, and detected using HRP-conjugated anti-mouse IgG and Immobilon Forte substrate. Chemiluminescent signals were visualized using an LAS-3000 mini imaging system.

Sodium phosphotungstic acid (Na-PTA) precipitation

Tissues (e.g., lymph nodes) were sectioned into 50 mg portions using 18G needles and homogenized in 1.5 mL Nippi Biomasher II tubes (Nippi, Tokyo, Japan). A digestion solution

containing 50 mM Tris-HCl (pH 7.5), 2% Triton X-100, 0.5% Sarkosyl, 100 mM NaCl, 5 mM MgCl2, 2 mM CaCl2, 0.5 mg collagenase, and 10 μg DNase I was added to each sample. Homogenates were incubated at 37°C for 2 hours with periodic vortexing. Following centrifugation at 20,000 x g for 30 minutes at 20°C, the supernatant was discarded, and the pellet was resuspended in 6.25% lauroylsarcosine. After centrifuging at 5,000 x g for 5 minutes, the supernatant was treated with Na-PTA to a final concentration of 0.3% and incubated at 37°C for 30 minutes with rotation. The sample was then centrifuged, washed with PBS, and resuspended in 20 μL of PBS for protein misfolding cyclic amplification (PMCA). To monitor potential contamination, tissue samples from both inoculated and uninoculated macaques were simultaneously prepared under identical conditions, provided uninoculated tissues were available.

Ethanol precipitation

A 10% spinal cord homogenate (50 μL) was mixed with 450 μL of ethanol, vortexed, and left to stand at room temperature for 5 minutes. The mixture was then centrifuged at 20,000 x g for 5 minutes, and the supernatant was transferred to a fresh tube. The pellet was resuspended in 500 μL of ethanol, vortexed, and incubated for another 5 minutes at room temperature. After a second centrifugation at 20,000 x g for 5 minutes, the supernatant was combined with the first. The pellet was air-dried and resuspended in 20 μL of PBS. The suspension was used for PMCA. To monitor potential contamination, spinal cord samples from both inoculated and uninoculated macaques were simultaneously prepared under identical conditions.

PMCA

The PMCA protocol was adapted from a previous report (*8*), with minor modifications. Ten percent (w/v) wild-type mouse BHs, containing 1 × PBS, 4 mM EDTA, and 1% Triton X-

100, were supplemented with digitonin (0.05%), heparin (300 µg/ml), arginine ethyl ester (10 mM), and 4-sulfotetrafluorophenyl ester (0.01%). This mixture served as the PrP^C substrate. PMCA was performed using an automatic cross-ultrasonic protein-activating apparatus (ELESTEIN 070-GOT; Elekon Science Corp., Chiba, Japan). Amplification was performed using 32 cycles of sonication (pulse oscillation for 3 seconds, repeated five times at the intervals of 0.1 seconds), followed by incubation at 37°C for 30 minutes. The initial PMCA product was further diluted to 1:5 with a fresh PrP^C substrate for subsequent rounds. PMCA products (2.5 µL) were digested with 40 µg/mL proteinase K at 37°C for 1 hour. Samples were boiled in SDS sample buffer for 5 minutes, separated via SDS-PAGE using 15% Tris-glycine gels, and electronically transferred onto polyvinylidene fluoride membranes. The membranes were probed with anti-PrP horseradish peroxidase-conjugated monoclonal antibody T2 (*9*).

We used brain homogenates from macaques intracerebrally inoculated with L-BSE (L-BSE IC macaque #23) and cattle intracerebrally inoculated with C-BSE, L-BSE, and H-BSE (C-BSE, L-BSE, and H-BSE IC cattle) as seeds for PMCA. The L-BSE IC macaque was second-generation macaques obtained by intracerebrally inoculating brain homogenates from a macaque that developed prion disease following inoculation with brain homogenates from natural Japanese L-BSE-affected cattle (JP24) (*3*). The C-BSE, L-BSE, and H-BSE IC cattle were experimentally inoculated with brain homogenates from C-BSE, L-BSE, and H-BSE-affected cattle at the National Institute of Animal Health, National Agriculture and Food Research Organization (NARO), Tsukuba, Ibaraki, Japan. To prevent contamination, PMCA substrates were prepared under rigorous prion-free conditions. To monitor potential contamination and the *de novo* generation of prions, negative controls containing uninoculated macaque tissues and non-seeded samples were included in each PMCA round. Additionally, to avoid the cross-contamination bovine L-BSE and C-BSE BHs, PMCA using macaque tissue samples as seeds

was performed separately from PMCA using bovine and macaque L-BSE and C-BSE BHs as seeds.

PK degradation assay

To compare the resistance of PMCA products to PK digestion, the products from six rounds of serial PMCA were digested with various concentrations of PK (50 to 5000 µg/ml) at 37°C for 1 hour. WB analysis was performed in four or three independent experiments. The average intensity of the PrPres signal in each sample was expressed as a percentage relative to that in the sample digested with 50 µg/ml of PK.

Results and Discussion

Among the PrPres with banding patterns similar to those of C-BSE-seeded PrPres propagated in various L-BSE-inoculated macaque tissues (Appendix Figure 3, panel A), those derived from the thymus and median nerve of macaque #19, as well as the thoracic cord of macaque #18 exhibited significantly reduced PK resistance compared to other PrPres and bovine C-BSE-seeded PrPres (Appendix Figure 3, panel B), suggesting that these PrPres are distinct from C-BSE-like PrPres. Each tissue of L-BSE PO macaques may produce multiple prion strains with distinct properties.

When the ileum sample from macaque #18 was used as a seed in PMCA using wild-type mouse BH as a substrate, distinct banding patterns of PrPres were amplified within the same sample (Figures 2 and 3). Therefore, it is suggested that two distinct PrP^{Sc} variants may exist in the ileum of #18 in minute amounts, and the amplification of either strain likely occurred by chance during PMCA, resulting in the selective propagation of one prion strain. In contrast,

when TgBo BH was used in PMCA, the C-BSE-like prion in the ileum of #18 appeared to be preferentially amplified as the seed.

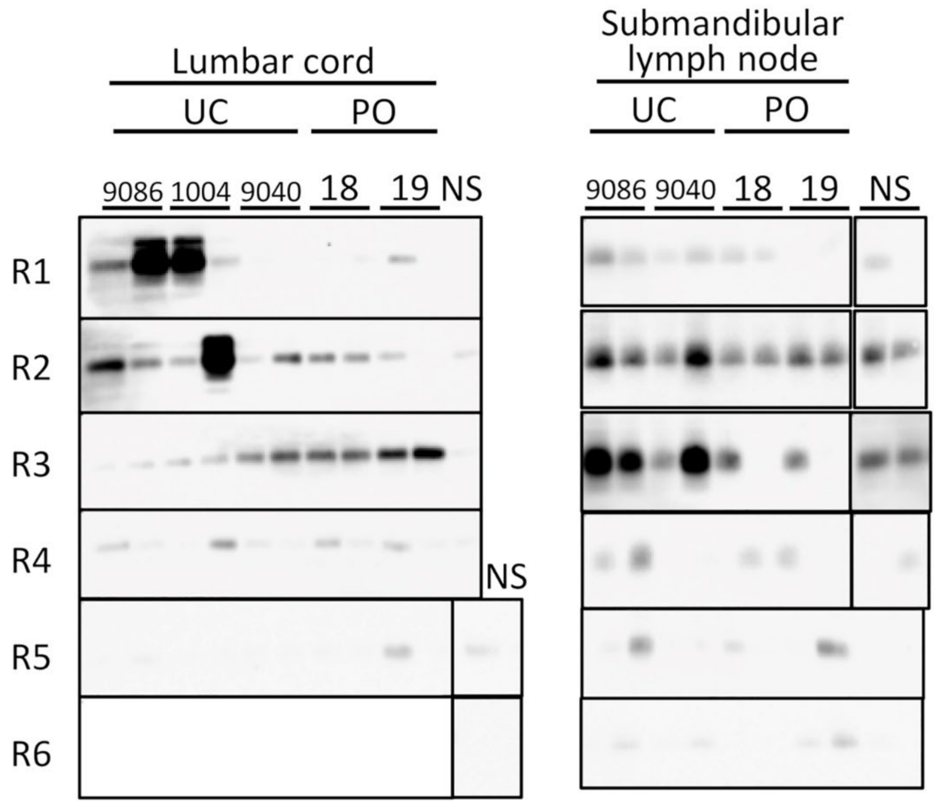

Appendix Figure 1. Proteinase K-resistant PrP^{Sc}-like prion protein was not amplified by PMCA in the lumbar cord or submandibular lymph nodes of both macaques (#18 and #19) orally inoculated with L-BSE prions. Sample preparation, PMCA, and western blotting were conducted as described in Figure 2. UC, uninoculated control; PO, L-BSE orally inoculated macaques.

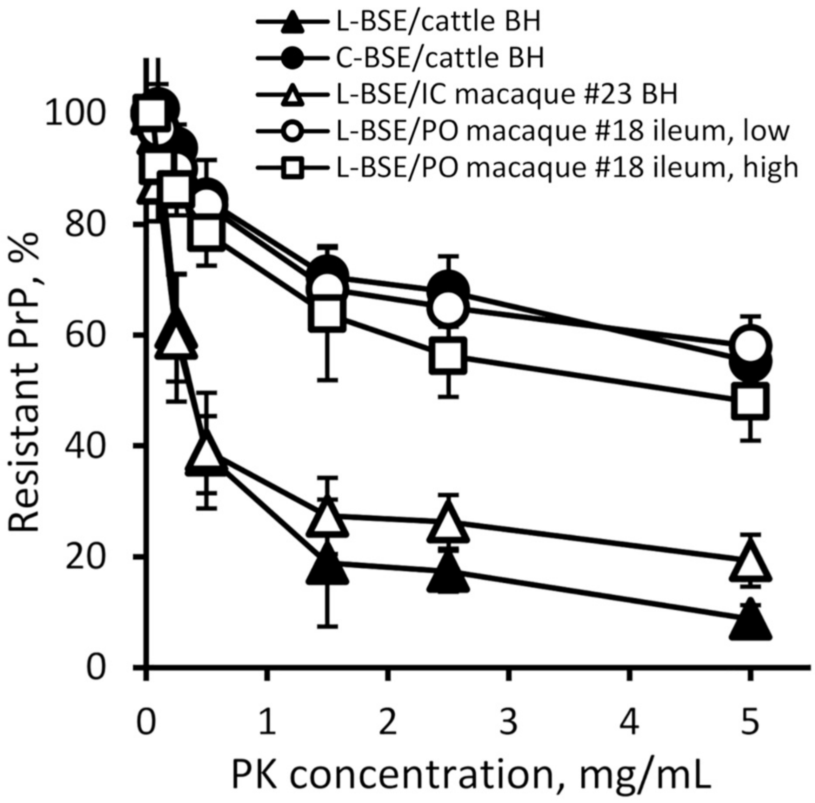

Appendix Figure 2. PK resistance of the 6th round PMCA products, seeded with ileum obtained from an L-BSE PO macaque #18 (low-type = open circle; high-type = open square), was compared with PK resistance of brains obtained from L-BSE/IC macaque #23 (open triangle), L-BSE–affected cattle (closed triangle), and C-BSE–affected cattle (closed circle). A PK degradation assay was performed with PK concentrations of 50 µg/mL–5 mg/mL. The PrPres signal intensity after each PK treatment is expressed relative to the western blot signal intensity of PrPres treated with 50 µg/mL PK, which is set as 100%. Error bars represent standard deviation. BH, brain homogenate, C-BSE, classical bovine spongiform encephalopathy; IC, inoculated intracerebrally; L-BSE, L-type bovine spongiform encephalopathy; PK, proteinase K; PrP, prion proteins; PO, inoculated orally.

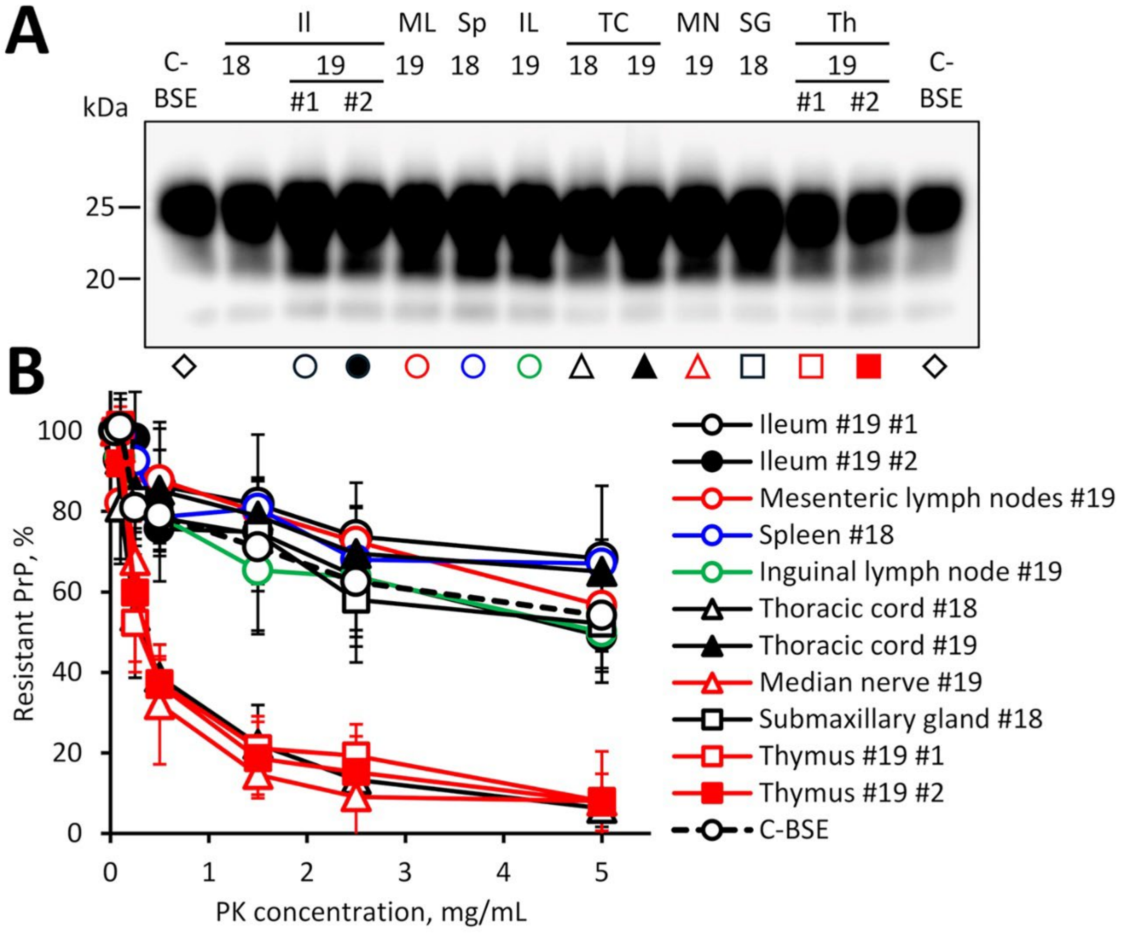

Appendix Figure 3. (A) Among the PrPres (proteinase K–resistant PrP^{Sc}-like prion proteins) amplified from various tissues of macaques orally inoculated with L-BSE, those exhibiting a banding pattern similar to that of PrPres obtained from PMCA using the brain homogenates of C-BSE-affected cattle as the seed were subjected to western blotting. The symbols correspond to the markers in panel B. In panel B, a PK degradation assay was conducted on the PrPres from panel A, following the method described in the Appendix. Remarkably, the PK resistance of the PrPres amplified from the thymus of macaque #19, the thoracic cord of macaque #18, and the median nerve of macaque #19 was significantly weaker than that of other PrPres, suggesting that these PrPres are distinct from C-BSE–like PrPres. Il (ileum), ML (mesenteric lymph nodes), Sp (spleen), IL (inguinal lymph nodes), PrP^{Sc}, abnormal prion proteins; TC (thoracic cord), MN (median nerve), SG (submaxillary gland), Th (thymus).
